# Efficacy of EUS-guided FNB using a Franseen needle for tissue acquisition and microsatellite instability evaluation in unresectable pancreatic lesions

**DOI:** 10.1186/s12885-020-07588-5

**Published:** 2020-11-11

**Authors:** Mitsuru Sugimoto, Hiroki Irie, Tadayuki Takagi, Rei Suzuki, Naoki Konno, Hiroyuki Asama, Yuki Sato, Jun Nakamura, Mika Takasumi, Minami Hashimoto, Tsunetaka Kato, Ryoichiro Kobashi, Yasuyuki Kobayashi, Yuko Hashimoto, Takuto Hikichi, Hiromasa Ohira

**Affiliations:** 1 Department of Gastroenterology, Fukushima Medical University, School of Medicine, 1 Hikarigaoka, Fukushima, 960-1295 Japan; 2 Department of Endoscopy, Fukushima Medical University Hospital, Fukushima, Japan; 3 Department of Diagnostic Pathology, Fukushima Medical University, School of Medicine, Fukushima, Japan

**Keywords:** EUS-FNA, EUS-FNB, Unresectable pancreatic lesion, Microsatellite instability

## Abstract

**Background:**

The efficacy of immune checkpoint blockade in the treatment of microsatellite instability (MSI)-high tumors was recently reported. Therefore, the acquisition of histological specimens is desired in cases of unresectable solid pancreatic lesions (UR SPLs). This study investigated the efficacy of endoscopic ultrasound-guided fine-needle biopsy (EUS-FNB) using a Franseen needle for UR SPL tissue acquisition and MSI evaluation.

**Methods:**

A total of 195 SPL patients who underwent EUS-guided fine-needle aspiration (EUS-FNA) or EUS-FNB (EUS-FNAB) between January 2017 and March 2020 were enrolled in this study. Among them, 89 SPL patients (FNB: 28, FNA: 61) underwent EUS-FNAB using a 22-G needle (UR SPLs: 58, FNB: 22, FNA: 36) (UR SPLs after starting MSI evaluation: 23, FNB: 9, FNA: 14).

**Results:**

The puncture number was significantly lower with FNB than with FNA (median (range): 3 (2–5) vs 4 (1–8), *P* <  0.01, UR SPLs: 3 (2–5) vs 4 (1–8), *P* = 0.036). Histological specimen acquisition was more commonly achieved with FNB than with FNA (92.9% (26/28) vs 68.9% (42/61), *P* = 0.015, UR SPLs: 100% (22/22) vs 72.2% (26/36), *P* <  0.01). The histological specimen required for MSI evaluation was acquired more often with FNB than with FNA (88.9% (8/9) vs 35.7% (5/14), *P* = 0.03).

**Conclusions:**

EUS-FNB using a Franseen needle is efficient for histological specimen acquisition and sampling the required amount of specimen for MSI evaluation in UR SPL patients.

## Background

Endoscopic ultrasound-guided fine-needle aspiration (EUS-FNA) is a safe and efficient procedure for diagnosing solid pancreatic lesions (SPLs), with a reported diagnostic sensitivity, specificity, and accuracy for SPL of 79–95.0%, 75.0–100%, and 78.0–96.0%, respectively [1–6]. Furthermore, EUS-guided fine-needle biopsy (EUS-FNB) using a TruCut needle improves the diagnostic yield and tissue acquisition and reduces the number of needle passes [7–9].

Microsatellite instability (MSI) is known as a predictive biomarker for the therapeutic effect of immune checkpoint blockade. Pembrolizumab has been proposed as a second-line treatment for any MSI-high cancer patients [10, 11]. In the guidelines for pancreatic cancer published by the Japan Pancreatic Society, it is stated that pembrolizumab is a second-line treatment for locally advanced or metastatic pancreatic cancer [12]. However, sufficient tissue preparation is needed to investigate MSI.

Recently, EUS-FNB using a Franseen needle has been reported to be more useful for tissue acquisition in cases of SPL [13–15]. However, tissue acquisition is not absolutely required for patients with resectable (R) SPL. Because tissue acquisition is important for MSI evaluation, tissue acquisition by EUS-FNB is required for patients with unresectable (UR) SPL. It remains unknown whether EUS-FNB using a Franseen needle is effective for tissue sampling and MSI evaluation only in UR SPL patients. Therefore, we aimed to reveal the efficacy of EUS-FNB using a Franseen needle in UR SPL patients.

## Methods

### Study design and ethics

This study is the retrospective study. Patients were not required to give informed consent because this study used anonymous clinical data obtained after each patient had agreed to medical activities by written consent. For full disclosure, the details of this study are published on the home page of Fukushima Medical University.

### Patients

**Fig. 1:**
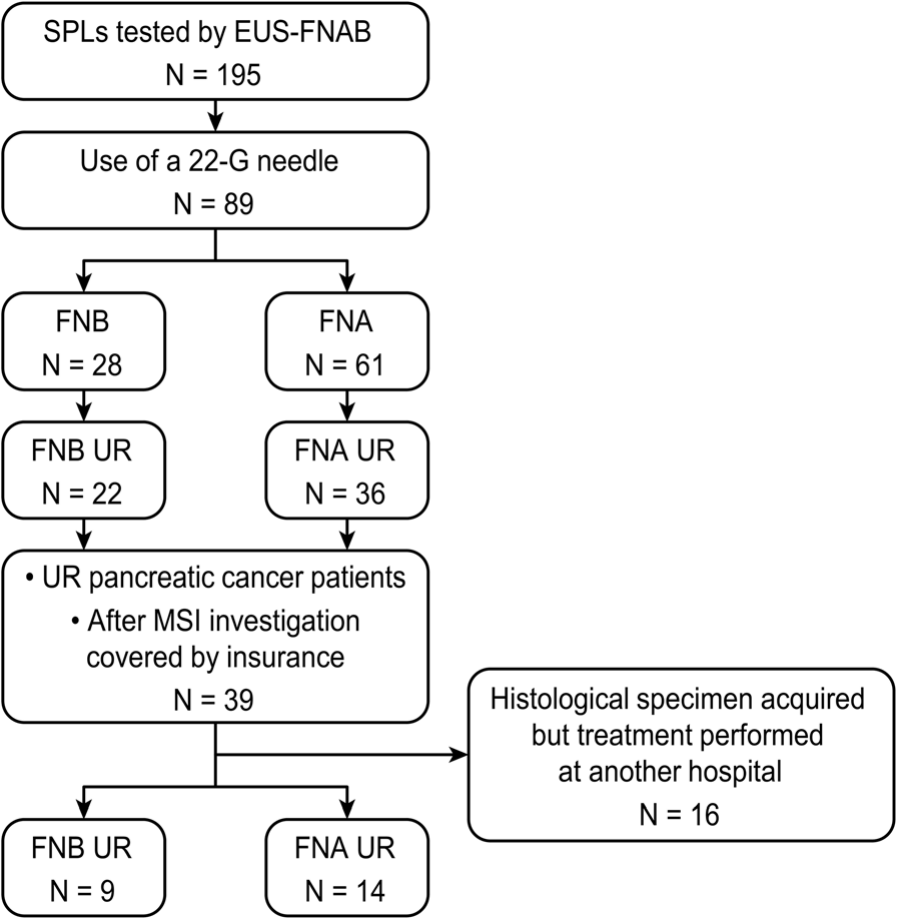
Flow chart of patient grouping in this study. *SPL*, solid pancreatic lesion; *EUS-FNAB*, endoscopic ultrasound-guided fine-needle aspiration biopsy; *UR*, unresectable; *MSI*, microsatellite instability

A total of 195 SPL patients who underwent EUS-FNA or EUS-FNB (EUS-FNAB) between January 2017 and March 2020 were enrolled in this study (Fig. 1). Among these patients, 89 SPL patients underwent EUS-FNAB using a 22-G needle (FNB: 28 patients, FNA: 61 patients). The SPLs of 58 patients were UR (FNB: 22 patients, FNA 36 patients).

Thirty-nine pancreatic cancer patients underwent EUS-FNAB after the MSI evaluation was covered by insurance in Japan. Among these patients, 16 patients had undergone the process of histological specimen acquisition but had received treatment at another hospital. Among the other 23 patients, EUS-FNB was performed in 9 patients, and EUS-FNA was performed in 14 patients.

### EUS-FNAB procedures

The echoendoscope was gently inserted into the patient while in the left lateral decubitus position after sufficient sedation by the intravenous administration of midazolam. After the SPL was visualized on the monitor, the lack of blood flow on the aspiration line was confirmed using Doppler mode. Then, the needle was advanced to the SPL, and the stylet was removed. The needle was passed back and forth 20 times in the SPL while applying suction using a 20- or 10-ml syringe. The fanning method was utilized as much as possible for puncture [16]. Aspiration was repeated until sufficient material was confirmed by rapid onsite cytology (ROSE) [17, 18].

**Fig. 2:**
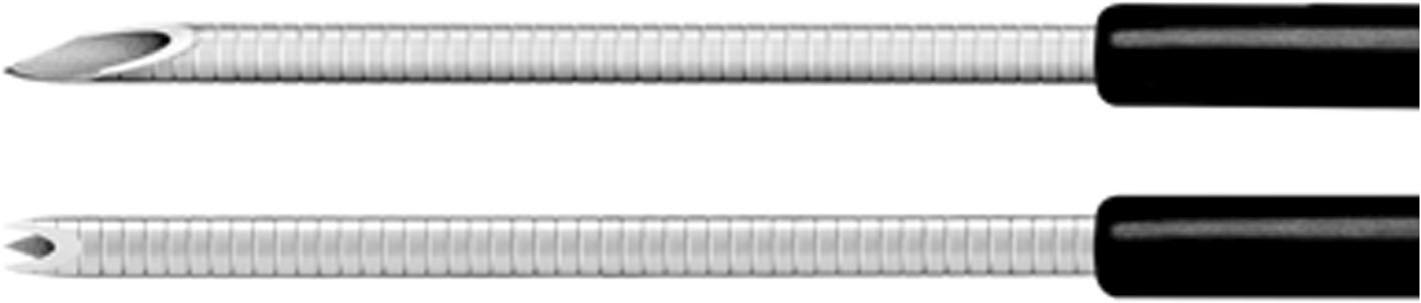
The Franseen needle used for EUS-FNB (image provided by Boston Scientific Japan). The needle on the top is a conventional EUS-FNA needle. The cutting surface of the conventional FNA needle is in a lancet shape. The needle on the bottom is an FNB needle. The cutting surface is larger by adopting the Franseen design

The following ultrasonography equipment and echoendoscopes were used in this study: EU-ME-1, EU-ME-2, GF-UC240AL-5, or GF-UCT260 (Olympus Medical Systems, Tokyo, Japan). The selection of the FNAB needle was as follows. For patients with an R SPL or a small SPL, a conventional FNA needle tended to be used because of its good penetration ability. Otherwise, the chosen needle was randomly selected by each endoscopist. The Franseen needle used for EUS-FNB in this study was an Acquire 22-G needle (Boston Scientific, MA, USA) (Fig. 2). The cutting surface of the Franseen needle (on the bottom of Fig. 2) is larger than that of the conventional aspiration needle (on the top of Fig. 2). The needles used for EUS-FNA in this study were an Expect 22-G needle (Boston Scientific, MA, USA), an EZ Shot 3 plus 22-G needle (Olympus Medical Systems), and an EchoTip 22-G needle (Cook Medical, Inc., NC, USA).

All procedures were performed by pancreaticobiliary specialists who had performed more than 200 EUS-FNAB procedures or beginners under the guidance of pancreaticobiliary specialists. The puncture needles were randomly chosen by each endoscopist.

### Examination items

The patient characteristics (age, sex), SPL data (tumor size, tumor location, resectability), and procedural characteristics (puncture route, puncture number, histology, adverse events) were compared between patients who underwent EUS-FNB and those who underwent EUS-FNA. In the UR SPL patients, similar comparisons were performed. The tumor size was the maximum diameter measured by EUS or computed tomography (CT). UR SPLs were determined by the following characteristics: 1. contacts/invades the superior mesenteric vein or portal vein by more than 180 degrees with a range of contact/invasion exceeding the inferior duodenal angle; 2. contacts/invades the superior mesenteric artery or celiac artery by more than 180 degrees; 3. contacts/invades the common hepatic artery with a range of contact/invasion extending to the proper hepatic artery or celiac artery; 4. contacts/invades the aorta; and 5. distant metastases [19, 20]. R SPLs were finally diagnosed by surgery. On the other hand, UR SPLs were diagnosed by cytology or histology on EUS-FNAB. Malignancy was defined by class IV or V cytology.

In some UR SPL patients who underwent EUS-FNAB after the MSI evaluation was covered by insurance, the possibility of performing the MSI evaluation using the histological specimens was compared between EUS-FNB and EUS-FNA.

### Requirements for histology and MSI evaluation

A histological diagnosis was attempted to be made using all the specimens submitted to department of pathology. To diagnose malignancy, the specimen must include tumor tissue that is not crushed or degenerated. If tumor tissues are fragmented or crushed, the diagnosis of malignancy become difficult. Furthermore, if a specimen contains inflammatory components, it become difficult to distinguish whether atypical cells are tumoral or reactive to inflammation.

To extract DNA for MSI evaluation, more than 2000 tumor cells are necessary. Additionally, more than 50% tumor cell content is needed. More than five unstained slide specimens of SPLs had to be prepared for the investigation of MSI. An MSI detection kit (FALCO HOLDINGS Co., Ltd., Kyoto, Japan) was used to measure MSI according to five mononucleotide markers (BAT-25, BAT-26, NR-21, NR-24, and MONO-27). When more than two markers were MSI positive, the tumor was defined as MSI-high.

### Statistical analyses

Continuous variables that did not conform to a normal distribution and ordinal variables were compared by the Mann-Whitney U test. Nominal variables were compared by Fisher’s exact test. A *P* value < 0.05 was considered significantly different. All statistical analyses were performed with EzR (Saitama Medical Centre, Jichi Medical University, Saitama, Japan) [21].

## Results

### FNAB with a 22-G needle in SPL patients

**Fig. 3:**
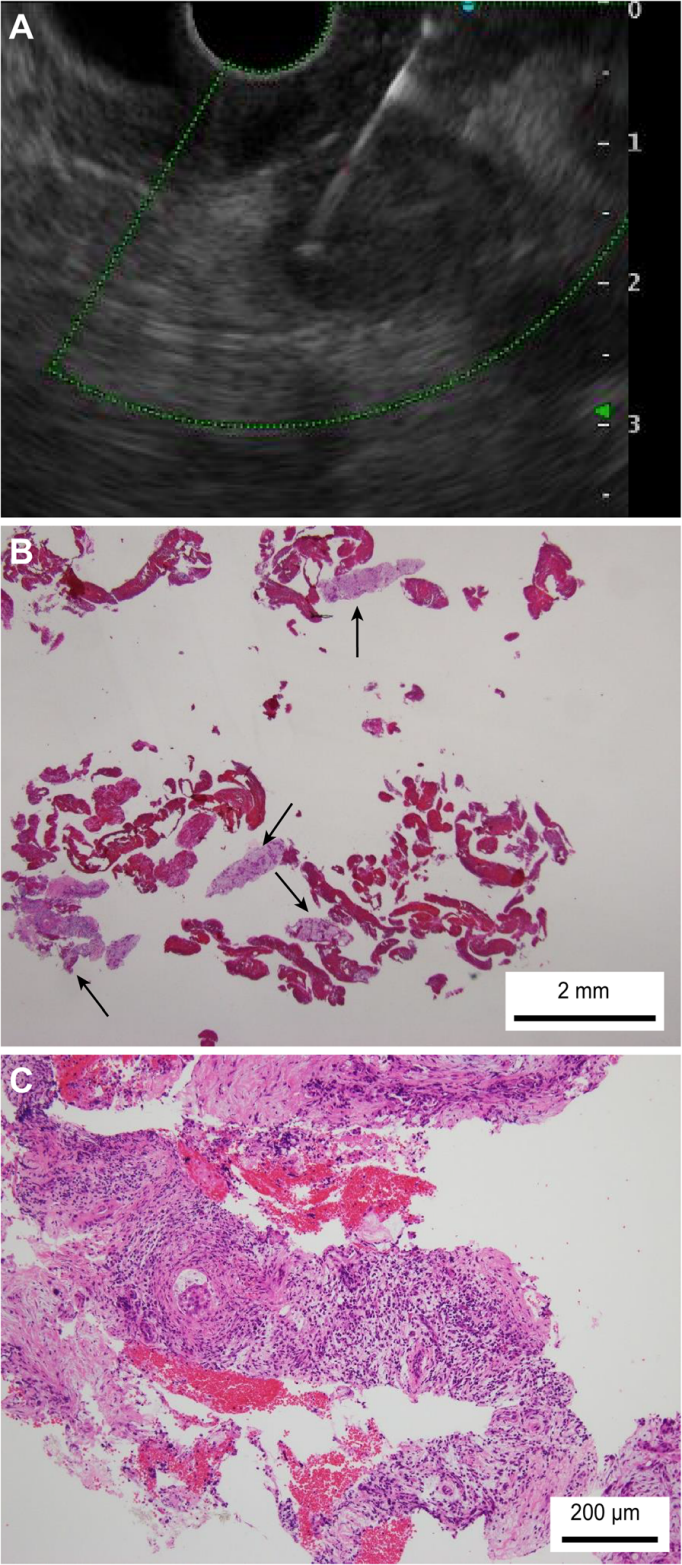
EUS-FNB specimen obtained using a Franseen needle. **a** A 15-mm pancreatic cancer lesion was punctured with a 22-G Franseen needle. **b** Some large tissue preparations were confirmed. (**c**) A sufficient number of tumor cells were observed

**Fig. 4:**
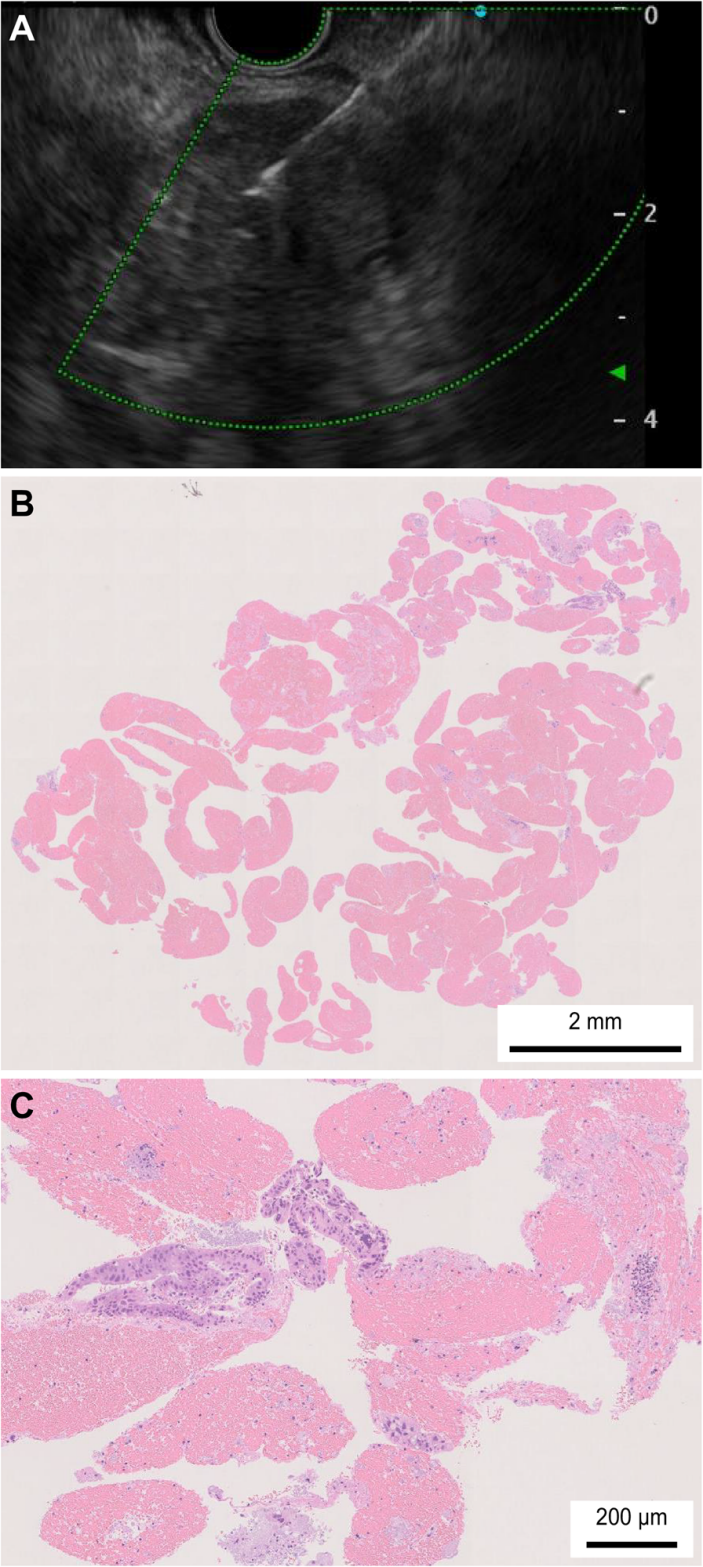
EUS-FNA specimen obtained using a lancet-shaped needle. **a** The visibility of the 22-G lancet-shape needle was not much different from that of the Franseen needle. **b** In a low-power field, evident large tissue preparations were not observed. **c** In a high-power field, tumor cells were confirmed; however, there were not as many as in Fig. 3c

The patient characteristics, final diagnosis, tumor size, tumor location, resectability, puncture route, and adverse events were not significantly different between patients who underwent FNB and those who underwent FNA (Table 1). Both adverse events after FNA improved with conservative treatment. The puncture number was significantly lower with FNB than with FNA (median (range); 3 (2–5) vs 4 (1–8), *P* value < 0.01). Furthermore, histological specimens were obtained significantly more often by FNB than by FNA (92.9% (26/28) vs 68.9% (42/61), *P* value = 0.015). A tissue specimen obtained by EUS-FNB is shown in Fig. 3. A tissue specimen obtained by EUS-FNA is shown in Fig. 4.

**Table 1:** Patient and SPL characteristics and outcomes of EUS-FNAB

	FNB (*N* = 28)	FNA (*N* = 61)	*P* value
Age, y, median (range)	68 (49–91)	70 (38–85)	0.33
Sex, male/female	18/10	30/31	0.25
Final diagnosis			0.09
Pancreatic cancer	28	54	
Pancreatic neuroendocrine tumor		7	
SPL size, mm, median (range)	25 (15–50)	27 (7–82)	0.81
SPL location, head/body or tail	11/17	27/34	0.82
Lesion resectability, R/UR	6/22	25/36	0.09
Puncture route, gastric/duodenal	20/8	42/29	1.0
Puncture number, median (range)	3 (2–5)	4 (1–8)	< 0.01
Histological specimen, n (%)	26 (92.9)	42 (68.9)	0.015
Adverse events, n (%)	0 (0)	2 (3.3)	1.0
Acute pancreatitis, n		1	
Bleeding, n		1	

*SPL* Solid pancreatic lesion, *R* Resectable, *UR* Unresectable

### FNAB with a 22-G needle in UR SPL patients

The patient characteristics, final diagnosis, tumor size, tumor location, puncture route, and adverse events were not significantly different between patients who underwent FNB and those who underwent FNA (Table 2). The puncture number was significantly lower with FNB than with FNA (median (range); 3 (2–5) vs 4 (1–8), *P* value = 0.036). Futhermore, histological specimens were obtained significantly more often by FNB than by FNA (100% (22/22) vs 72.2% (26/36), *P* value < 0.01).

**Table 2:** Patient and SPL characteristics and outcomes of EUS-FNAB in UR SPL patients

	FNB, UR (*N* = 22)	FNA, UR (*N* = 36)	*P* value
Age, y, median (range)	68 (49–91)	70 (38–84)	0.79
Sex, male/female	15/7	21/15	0.58
Final diagnosis			0.52
Pancreatic cancer	22	34	
Pancreatic neuroendocrine tumor		2	
SPL size, mm, median (range)	30 (15–50)	30 (10–82)	0.70
SPL location, head/body or tail	8/14	14/22	1.0
Puncture route, gastric/duodenal	16/6	26/10	1.0
Puncture number, median (range)	3 (2–5)	4 (1–8)	0.036
Histological specimen, n (%)	22 (100)	26 (72.2)	< 0.01
Adverse events, n (%)	0 (0)	1 (2.8)	1.0
Bleeding, n		1	

*SPL* Solid pancreatic lesion, *UR* unresectable

### MSI of pancreatic cancer specimens obtained by EUS-FNAB

The size of the SPL, puncture route, and number of punctures were not significantly different between patients who underwent FNB and those who underwent FNA (Table 3). The MSI evaluation was achieved significantly more often with FNB than with FNA (88.9% (8/9) vs 35.7% (5/14), *P* value = 0.03). MSI-high tumors were not observed in any of the patients.

**Table 3:** Comparison of MSI

	FNB, UR (*N* = 9)	FNA, UR (*N* = 14)	*P* value
SPL size, mm, median (range)	25 (15–50)	30 (15–82)	0.63
Puncture route, gastric/duodenal	7/2	9/5	0.66
Puncture number, median (range)	3 (2–5)	4 (1–7)	0.26
Possibility of MSI evaluation, n (%)	8 (88.9)	5 (35.7)	0.03
MSI-high tumors, n	0	0	

*MSI* Microsatellite instability, *SPL* Solid pancreatic tumor

## Discussion

In this manuscript, we investigated the efficacy of EUS-FNB using a Franseen needle for UR SPL patients. As a result, histological specimens were collected significantly more often with EUS-FNB than with EUS-FNA. Furthermore, EUS-FNB required fewer punctures than EUS-FNA for diagnosing UR SPLs, and the MSI evaluation was achieved more often by EUS-FNB than by EUS-FNA.

Several factors that may affect the diagnostic value of EUS-FNA have been reported. Regarding the relationship between EUS-FNA and tumor size, there are multiple points of view [5, 22]. Haba et al. [22] reported that in cases of smaller lesions, the diagnostic value of EUS-FNA is reduced. On the other hand, Uehara et al. [5] found that the tumor size does not influence the diagnostic value of EUS-FNA. It is true that EUS-FNA is difficult for small lesions; however, it is not true that the diagnostic value of EUS-FNA increases with increasing tumor size because the presence of necrosis could affect the accuracy of EUS-FNA. Contrast-enhanced EUS-FNA was reported to avoid puncture of necrotic areas [23–25]. Necrotic areas can be observed more clearly in larger SPLs than in small SPLs, and it is difficult to identify necrotic areas using only B-mode imaging. In this study, the UR SPLs were larger than the R SPLs (median (range): 30 (10–82) mm vs 20 (7–40) mm, *P* value < 0.01). Although the evaluation of necrotic areas on EUS is difficult, the superiority of EUS-FNB using a Franseen needle over EUS-FNA was proven in SPLs and UR SPLs.

In past reports, the frequency of MSI-high tumors was 0–29% in pancreatic cancer [26–31]. However, frequencies of MSI-high tumors of more than 10% were reported in small studies. In a study with a large number of patients (*n* = 3954) [28], MSI-high pancreatic cancer was found in very few patients (0.5%). In a study that targeted 12,019 patients with 32 types of cancer, the rate of MSI-high tumors was less than 2% among those with pancreatic cancer [32]. Therefore, it is reasonable that MSI-high tumors were not observed in this study.

What about the difference in the measurement method? In recent reports using next-generation sequencing, the frequency of MSI-high tumors among patients with pancreatic cancer was 0.5% or less than 2% [28, 32]. On the other hand, Yamamoto et al. [30] reported that 13% of pancreatic cancer patients showed MSI-high tumors according to PCR and that the results were satisfactory. In the report written by Yamamoto et al. [30], the Bethesda panel was used. In this study, the Promega panel was used. In the report written by Murphy et al. [33], the Promega panel was superior to the Bethesda panel. Therefore, there should be no problem with the MSI measurement method in this study.

As described above, the frequency of MSI-high tumors is low in pancreatic cancer. However, dramatic efficacy of pembrolizumab has been reported in MSI-high pancreatic cancer [31]. Thus, EUS-FNB using a Franseen needle should be performed to collect sufficient specimens for MSI evaluation. The shape of the needle tip could contribute to the superiority of FNB for tissue acquisition (Fig. 2). There are three cutting surfaces on a Franseen needle, whereas there is only one lancet-shaped cutting surface on a conventional FNA needle tip. This difference in the number of cutting surfaces may produce a difference in the cutting area.

Based on the above, we describe appropriate cases in which EUS-FNB should be performed. For diagnosing R SPLs, cytology alone is enough. Therefore, EUS-FNA is also sufficient for diagnosing these cases. However, for diagnosing UR SPLs, EUS-FNB should be performed to obtain a sufficient specimen for MSI investigation. Furthermore, EUS-FNB should be performed in patients whose disease requires many specimens for diagnosis, such as those with autoimmune pancreatitis [34, 35].

There are several limitations to this study. First, this study was a small retrospective study performed at a single institution. The types of puncture needles used were not randomly assigned. Therefore, selection bias could exist in this study. In the future, it is desirable for a multicenter prospective study to prove the argument of this study. Second, the types of SPLs were not unified. However, only UR pancreatic cancer patients were involved in the MSI comparison. Therefore, there was no influence of the SPL type in the MSI comparison between FNB and FNA. Third, the weight and volume of cells were not measured. Instead, the suitability of histological specimen sampling was compared between the two groups. The histological evaluation was performed by experienced pathologists.

## Conclusions

EUS-FNB using a Franseen needle could be efficient for histological specimen acquisition and MSI evaluation in UR SPL patients.

## Data Availability

The datasets generated and/or analyzed during the current study are available from the corresponding author upon reasonable request.

## Abbreviations

MSI: Microsatellite instability
UR: Unresectable
SPL: Solid pancreatic lesion
EUS-FNB: EUS-guided fine needle biopsy
EUS-FNA: EUS-guided fine needle aspiration
EUS-FNAB: EUS-FNA or EUS-FNB
R: Resectable
ROSE: Rapid onsite cytology
CT: Computed tomography
